# Ultramicroporous Polyphenylenes via Diels–Alder Polycondensation Approach

**DOI:** 10.3390/polym15092060

**Published:** 2023-04-26

**Authors:** Svetlana A. Sorokina, Nina V. Kuchkina, Alexander V. Mikhalchenko, Irina Yu. Krasnova, Dmitry A. Khanin, Kirill M. Skupov, Zinaida B. Shifrina

**Affiliations:** A.N. Nesmeyanov Institute of Organoelement Compounds, Russian Academy of Sciences, 28 Vavilov St., 119991 Moscow, Russia; kuchkina@ineos.ac.ru (N.V.K.);

**Keywords:** porous organic polymers (POPs), microporous non-network polymers, polyphenylenes, Diels–Alder reaction, solution processability

## Abstract

Development of new microporous organic polymers attracts significant attention due to a wide scope of promising applications. In addition, the synthesis of soluble, non-crosslinking polymers of high surface area and uniform microporosity is very challenging, and the methods for soluble microporous polymers formation are rather limited. In this work, we report a new approach to construct porous polyphenylenes which employs the Diels–Alder polycondensation of multifunctional ethynyl-containing monomers of different spatial architecture with bis(cyclopentadienone)s. The resulting polymers were soluble in common organic solvents, and their structure and properties were assessed by NMR, TGA, DSC, and SEC studies. The polymers demonstrated a specific surface area up to 751 m^2^·g^−1^ and ultramicroporous (pore size ≤ 0.6 nm) structure. N_2_ and CO_2_ adsorption–desorption data revealed that porosity parameters, e.g., specific surface area and pore sizes, can be tuned selectively by varying the type of monomers and reaction conditions.

## 1. Introduction

Design of new organic microporous polymers has gained considerable interest over the last two decades due to a vast diversity of potential applications, including gas capture and separation [1,2,3,4], photovoltaics [5,6], chemical sensing [7,8], and catalysis [9,10,11,12,13,14]. These materials are characterized by high specific surface area (SSA), which arises from the voids presented within the polymer architecture and formed during the synthesis due to specific structure of macromolecules and their arrangement. There are two tentatively distinguished approaches to the formation of microporous materials. The general synthetic strategy involves the network formation due to crosslinking of rigid monomers by robust covalent or strong coordination bonds. Crystalline metallic organic frameworks (MOFs) and covalent organic frameworks (COFs), as well as amorphous porous aromatic frameworks (PAFs), conjugated microporous polymers (CMPs), hyper crosslinked polymers (HCPs), etc., are the porous organic polymers (POPs) whose high SSA (up to 6000 m^2^/g) have been obtained due to network formation [15,16,17]. However, these compounds are insoluble in organic solvents, which significantly hampers their processability and limits the areas of application.

Another approach implies the formation of microporosity due to inefficient packing of highly rigid and contorted polymer chains. The intermolecular interactions in such polymers are precluded due to distorted structure of the monomers, which leads to the formation of interconnected voids and emergence of free volume. The process occurs without network formation. Therefore, the resulting polymers are soluble in common organic solvents [18]. The weak cohesion inter-chain interactions and free volume accessible for solvent molecules also contribute to the solubility of these polymers. This type of POP is characterized by moderate SSA (100–1000 m^2^/g), with the fraction of micropores usually reaching 50%.

The key advantage of the non-networking POPs is their solution processability. This enables the preparation of different materials, e.g., the feasibility in film casting, commercial roll-to-roll fabrication, membrane formation, etc., [3,8,18,19,20,21,22]. Soluble POPs also may be employed as templating molecules in the synthesis of metal nanoparticles (NPs) [9,12]. Here, the solubility of non-networking POPs offers the advantages of NPs synthesis via thermal decomposition of metal precursors in the polymer solution over the wet impregnation method since more parameters could be finely tuned during the synthesis to afford the well-defined, uniform NPs with predicted size and morphology. The resulting nanocomposites are now of particular interest for catalysis [11,12,23,24,25]. The presence of micro- and mesopores restricts the growth of NPs within the polymer matrix while allowing the easy access and diffusion of substrate molecules in the resulting catalytic composites. In contrast to MOF-based catalysts, whose hydrolytically unstable bonds limit the reaction scope where they can be applied, the exceptional thermo-, chemo-, and hydrostability of POPs allows these materials to be used under harsh reaction conditions, including high water vapor pressure, acidic or alkaline environments, etc. [11,12].

The ease of functionalization, unique topologies, extraordinary stability, and solution processability of non-networking POPs boost the research interest in expanding the chemistry underlying the non-networking POPs synthesis. Along with spirobisindane and polybenzodioxin [20,26], triptecene [27,28,29], and porphirine-based [25] monomers that are widely established structural units in the synthesis of soluble microporous polymers so-called polymers of intrinsic microporosity (PIMs), many other building blocks, such as phenantroline [30], benzylamine [31], bisphosphine [32], and phtalocyanine [33], have been utilized to create microporosity in polymers. Phenylenes of different structures are another class of compounds capable of forming micropores. The general approaches to the synthesis of porous polyphenylenes (PPPhs) are based on the crosslinking of aromatic building blocks, such as Suzuki cross-coupling of 1,2,4,5-tetrabromobenzene and 1,4-benzene diboronic acid, Friedel–Crafts alkylation, or trimerization of ethynyl groups [34,35,36,37,38]. The resulting compounds are characterized by the moderate-to-high surface areas in the range of 470–980 m^2^/g. The average micropore surface area constitutes half of the total SSA, while certain chemical modifications may increase this value up to 80% [36,37]. PPPhs are the attractive macromolecules due to ease of post-modification, the possibility of introducing of different functional groups, and exceptional thermal properties. They can be used as semiconductors, light harvesting molecules, and gas separation membranes, exhibiting high gas selectivity for H_2_ relative to CO_2_, CO, and CH_4_ [34,35,39,40].

However, the aforementioned approaches allow the synthesis of insoluble crosslinking microporous materials. In the present study we applied the Diels–Alder polycondensation reaction to synthesize soluble porous polyphenylenes. It is known that the reactions used for POPs synthesis should provide more than one covalent bond for each structural unit [18]. In this regard, the Diels–Alder reaction seems to be a promising tool for the synthesis of soluble POPs via step-growth polycondensation of multitopic aromatic monomers, yielding a new arene ring at each condensation step and leading to hyperbranched macromolecular architecture. However, despite the applicability for the synthesis of some structural units which are suitable for production of polymers with high SSA, the Diels–Alder reaction has not yet been explored for the fabrication of POPs.

In this work, we used two multitopic monomers possessing different geometries, namely tetrakis(4-ethynylphenyl)methane and 1,3,5-triethynylbenzene, as the structural units for PPPhs synthesis by the Diels–Alder polycondensation reaction with bulky phenylene-substituted bis(cyclopentadienone)s. Previously, the aforementioned ethynyl-containing compounds were used in the POP synthesis by Sonogashira–Hagihara cross-coupling and Yamamoto polymerization reactions, yielding crosslinked insoluble products [15,17,41,42]. Our approach results in soluble polyphenylenes with SSA up to 751 m^2^/g, which could be effectively controlled during the synthesis.

## 2. Materials and Methods

### 2.1. Materials

Tetraphenylmethane (95%, ABCR, Karlsruhe, Germany), 1,3,5-tribromobenzene (98%, Acros Organics, Geel, Belgium), bromine (99%, Acros Organics, Geel, Belgium) trimethylsilylacetylene (98%, ABCR, Karlsruhe, Germany), CuBr (98%, Sigma-Aldrich, Darmstadt, Germany), CuI (98%, Fluka, Dresden, Germany), tetrakis(triphenylphosphine)palladium(0) (99%, Sigma-Aldrich, Darmstadt, Germany), bis(triphenylphosphine)palladium(II) dichloride (98%, Sigma-Aldrich, Darmstadt, Germany), Bu4NF (1M solution in THF, ABCR, Karlsruhe, Germany), bis(4-bromophenyl)ether (99%, Sigma-Aldrich, Darmstadt, Germany), 1,4-diiodobenzene (98%, ABCR, Karlsruhe, Germany), phenylacetylene (98%, Acros Organics, Geel, Belgium), 1,3-diphenyl-2-propanone (99%, Sigma-Aldrich, Darmstadt, Germany), potassium hydroxide (99%, Acros Organics, Geel, Belgium), glacial acetic acid (99%, Sigma-Aldrich, Darmstadt, Germany), potassium permanganate, triethylamine (99%, Acros Organics, Geel, Belgium), toluene (99.5%, Component Reactive, Moscow, Russia), ethanol (96%, Component Reactive, Moscow, Russia), diphenylether (99%, ABCR, Karlsruhe, Germany), THF ( HPLC grade, Carlo Erba reagents, Milan, Italy), and gases: N_2_ (99.999%) and CO_2_ (99.999%) were used as received.

### 2.2. Measurements

^1^H and ^13^C NMR spectra were recorded on the Avance IIIHD-500 MHz NMR spectrometer (Bruker, Bremen, Germany) operating at 500.13 MHz for ^1^H and at 125.76 MHz for ^13^C. Chemical shifts are given in parts per million (ppm), using the solvent signal as a reference. CD_2_Cl_2_ was used as solvent for all NMR measurements (δ(^1^H) = 5.35 ppm; δ(^13^C) = 53.4 ppm).

Thermogravimetric analysis (TGA) was carried out on a Shimadzu DTG-60H (Shimadzu GmbH, Kyoto, Japan) at a heating rate of 10°/min in argon.

Differential scanning calorimetry (DSC) measurements were performed on a DSC-3 (Mettler Toledo, Zurich, Switzerland) at a heating rate of 10°/min in argon.

Size-exclusion chromatography (SEC) analyses were carried out using a Shimadzu LC-20AT chromatograph (Shimadzu GmbH, Kyoto, Japan ) equipped with PSS SDV analytical columns 100 Å and 100,000 Å (particle size 10 μm, 8 × 300 mm). Detection was achieved with refractive index detector. SEC was performed in THF at a flow rate of 1.0 mL min^−1^, with polystyrene as a standard.

N_2_ and CO_2_ adsorption–desorption isotherms were obtained at 77 and 273 K, respectively, with the Surface Area and Pore Size Analyzer System 3P Micro 200 (3P Instruments GmbH & Co. KG, Odelzhausen, Germany) in the range of 10^−3^–1 bar. Before the measurements, the samples were degassed at 353 K for 24 h until the residual pressure reached 5 Pa. The Brunauer–Emmett–Teller (BET) equation was applied to the nitrogen adsorption isotherm data according to the Rouquerol criteria [43]. N_2_ cross-sectional area A and adsorbed N_2_ density ρads were taken as 0.162 nm^2^ and 0.808 g·mL^−1^, respectively. Non-linear density functional theory (NLDFT) method was applied to the carbon dioxide adsorption isotherm data using NovaWin, version 11.04, Quantachrome Instruments, considering CO_2_ cross-sectional area A of 0.210 nm^2^, adsorbed CO_2_ density ρads of 1.044 g mL^−1^, saturated vapor pressure of the adsorbate p0 of 3.485 MPa, and affinity coefficient β of 0.35 [44]. Pore size distributions and pore volumes were obtained according to the NLDFT method.

### 2.3. Synthetic Procedures

3,3′-(1,4-Phenylene)bis(2,4,5-triphenylcyclopenta-2,4-dien-1-one) (B2_1_) and 3,3′-(oxydi-1,4-phenylene)-bis(2,4,5-triphenylcyclopenta-2,4-dien-1-one) (B2_2)_ were synthesized as described in [45].

Tetrakis(4-ethynylphenyl)methane (A4) was synthesized according to the reported procedure [46]. 1,3,5-Triethynylbenzene (A3) was synthesized as described in the literature [47].

General Procedure for the Synthesis of PPPhs: All procedures were performed using a Schlenk flask under argon atmosphere in diphenyl ether at 165 °C using total monomer concentrations of 0.01 or 0.05 mol L^−1^, at equifunctional molar ratios of monomers.

In the typical procedure, a 50 cm^3^ Schlenk flask was charged with the bis-cyclopentadienone (B2) (0.168 mmol), tetrakis(4-ethynylphenyl)methane (A4) (0.084 mmol), or 1,3,5-triethynelbenzene (A3) (0.112 mmol) and diphenyl ether (amount for chosen concentration). The reaction was carried out for the desired time (see Table 1) at 165 °C, with stirring, in argon atmosphere, and then the mixture was allowed to cool, followed by precipitation into ethanol. The crude polymer was purified by multiple reprecipitation from chloroform into ethanol and dried in vacuo at 80 °C for 24 h. The reaction conditions and molecular weights of polymers prepared are shown in Table 1.

## 3. Results and Discussion

### 3.1. Synthesis and Characterization of PPPhs

**Figure 1:**
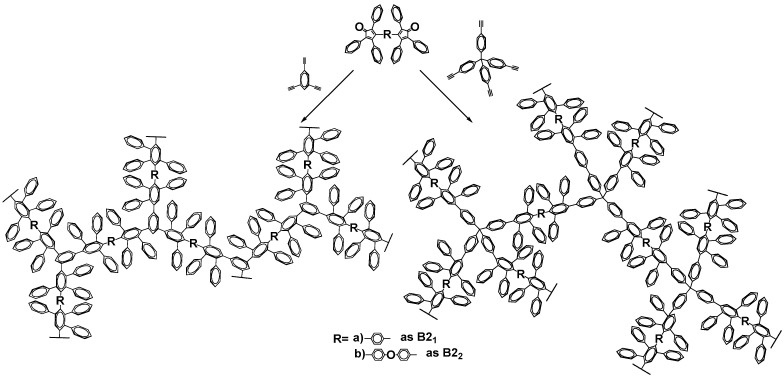
Proposed structures of polyphenylenes based on multifunctional monomers by Diels–Alder polycondensation.

Polyphenylenes were synthesized via Diels–Alder polycondensation reaction using two pairs of monomers (A4 + B2) and (A3 + B2) in diphenyl ether as a solvent (Figure 1). Phenyl substituted bis(cyclopentadienone)s were used as B2 monomers, and tetra- or tri-ethynyl containing compounds, namely tetrakis(4-ethynylphenyl)methane and 1,3,5-triethynylbenzene, as A4 and A3 monomers, respectively, were used as multibranching units. B2 monomers differed in the central structural group to estimate the influence of chemical composition on the surface area in molecular level, while A3 and A4 differed in spatial geometry of the molecule, namely trigonal pyramidal (A3) and tetrahedral (A4), which further implemented a macromolecular growth in different directions.

It is well-known that the polycondensation reaction of the multifunctional monomers is accompanied with the fast gelation due to the formation of polymer network. Since the aim of this work is the synthesis of non-crosslinking polymers, the reaction conditions, such as temperature, concentration of the monomers, and reaction time, were carefully adjusted to afford the formation of soluble macromolecules and to avoid the undesired crosslinking. The synthesis conditions and some characterization data are presented in Table 1. All the polymers were synthesized at equifunctional ratios of monomers, i.e., A4:B2 = 1:2 for tetrahedral A4 monomer and A3:B2 = 2:3 for trigonal A3. The molar concentration of the monomers dramatically impacts the formation of soluble polymer, and the polymer growth without fast gelation occurred in relatively dilute solution only. The effect was more pronounced for A4, where the soluble polymers were obtained at total a monomer concentration of 0.01 mol·L^−1^ and below. For polymers based on A3, no gelation was observed at 0.05 mol·L^−1^ for B2_2_ monomer, while the reaction with more rigid B2_1_ was more sensitive to an increase in molar concentration, yielding the insoluble product. The optimum temperature of synthesis was found to be 165 °C. The further increase in temperature led to formation of high fraction of crosslinking polymer along with soluble product. The reaction time influenced mainly the molecular weight of the resulted polymers, and no noticeable effect on the crosslinking process was observed at appropriate concentrations of the monomers.

The molecular weight of the polymers was determined by SEC. Some examples of SEC chromatograms are presented in Figure S1. Depending on the reaction conditions, the apparent molecular masses were in the range of 16–35 kDa for polymers based on tetrahedral monomer A4. For A3 monomer, the use of B2_2_ provided the product with molecular weight up to 789 kDa. It should be noted that the use of a pair of monomers, A3 and B2_2_, allowed the synthesis at higher concentration in comparison with the other pairs of monomers. Obviously, the increase in molar concentration dramatically affected the molecular weight of the polymers. Moreover, as can be seen from Table 1, the use of B2_2_ allows the formation of polymers of higher molecular weight for both A3 and A4 compared with B2_1_, apparently due to the greater reactivity of B2_2_.

The obtained polymers were powders of a beige color. The PPPhs were achieved with high yields. The PPPhs showed good solubility in common organic solvents, such as tetrahydrofuran, chloroform, dichloromethane, toluene, o-xylol, etc.

The structure of the polymers was assessed by ^1^H and ^13^C NMR spectroscopy. Figure S2a shows the ^1^H NMR spectrum of Polymer 2 based on A4 and B2_1_ bearing phenylene bridging group. The spectrum contains a set of signals related to aromatic protons at 6–8.0 ppm, which are broad and overlapping. This is consistent with the structure of the polymer composed of phenylene units. However, a signal at 7.63 ppm is clearly distinguished and ascribed to the protons of pentaphenyl-substituted benzene rings, which are newly formed during the Diels–Alder polycondensation. A weak signal at 3.17 ppm corresponds to the protons of the ethynyl groups. Obviously, the observed ethynyl groups originate from the incomplete Diels–Alder reaction of multitopic ethynyl-containing monomer. According to the integral ratio of the signals of ethynyl proton and protons of pentasubstituted benzene, their ratio is 1:14. This means that, on average, one ethynyl group is preserved in two repetitive units of polymer, and the structure can be presented as depicted in Figure S2b. The macromolecule growth occurred mainly in four directions; however, one ethynyl group per two tetraphenylmethane units remained unreacted. The results are in accordance with previous findings obtained for hyperbranched polypyridylphenylenes [48], which were synthesized by A6 and B2 Diels–Alder polycondensation. In that case, it was established that the growth of the most branched polymer has proceeded in five out of six possible directions. In the ^13^C NMR spectrum (Figure S2c), more signals can be distinguished. In particular, the peak at 63.95 ppm is ascribed to the tetrasubstituted carbon atom of the tetraphenylmethane unit. The low intensity peak at 200 ppm corresponds to the carbonyl groups of bis(cyclopentadienone), which are presented as terminal groups of the polymer. The carbon atoms of the acetylene groups are observed at 77.65, 78.16, and 83.17 ppm.

The ^1^H NMR spectrum of PPPhs based on the trifunctional unit (Figure S3a) is similar to that described above. However, the signal attributed to pentasubstituted phenylene moieties is shifted to a high field region and overlapped with the other signals, making reliable quantification of acetylene groups impossible. The ^13^C NMR spectrum (Figure S3b) confirms a very low content of ethynyl groups.

The thermal behavior of porous polyphenylenes were examined by TGA under nitrogen atmosphere and by DSC. As shown in Figure S4, the synthesized PPPhs exhibited exceptional thermal stability. The 5% weight loss was detected when the temperature was above 510 °C for all the samples. The results suggest that PPPhs are promising materials for high-temperature applications.

DSC results (Figure S5) determined that PPPhs are the glassy polymers, with glass transition temperature above the decomposition temperature. The results confirmed the exceptional rigidity of the synthesized structures that is a necessary feature of the high free-volume polymers.

### 3.2. Gas Adsorption Properties of PPPhs

**Figure 2:**
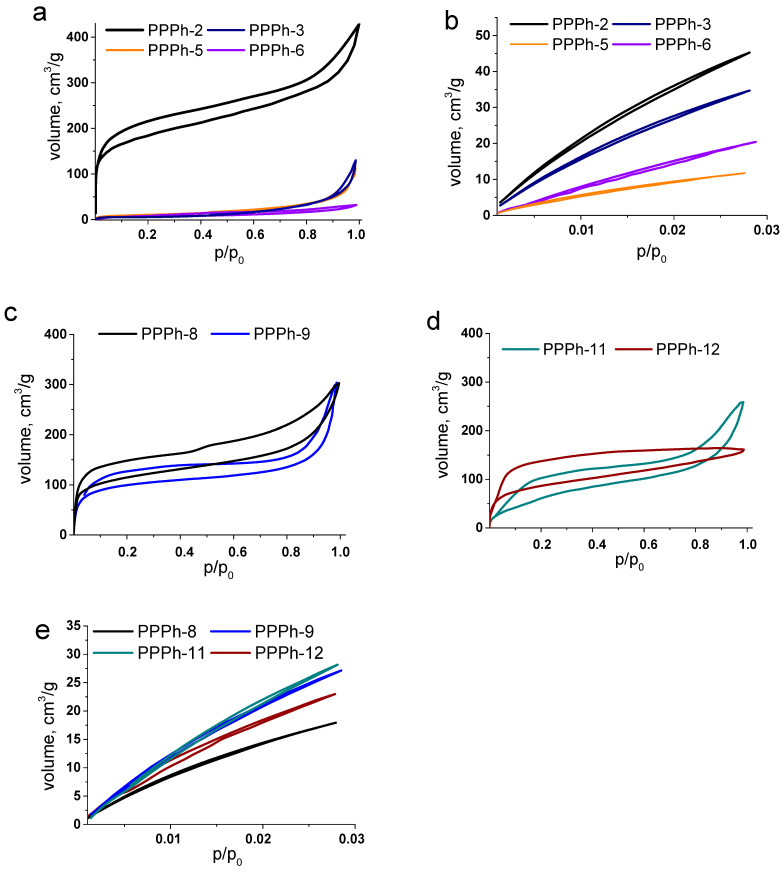
The nitrogen adsorption and desorption isotherms of PPPhs measured at 77 K (**a**,**c**,**d**). CO_2_ adsorption and desorption isotherms of PPPhs measured at 273 K (**b**,**e**).

The porous properties were investigated by N_2_ and CO_2_ adsorption–desorption measurements. Figure 2a,c,d show the nitrogen adsorption isotherms for tetra-branched and tri-branched polymers, respectively, and Figure 2b,e depict CO_2_ isotherms. Similar to other POPs, PPPhs demonstrated the hysteresis loops, which are often ascribed to the swelling of the polymer molecule in the presence of sorbate. Another possible explanation is the elastic deformation of the material which could arise at the desorption step. As a result, the penetrant molecule is captured in the matrix [18,49]. Nevertheless, similar behavior is often observed for microporous materials, especially for those having a slit-pore geometry [18,50].

**Figure 3:**
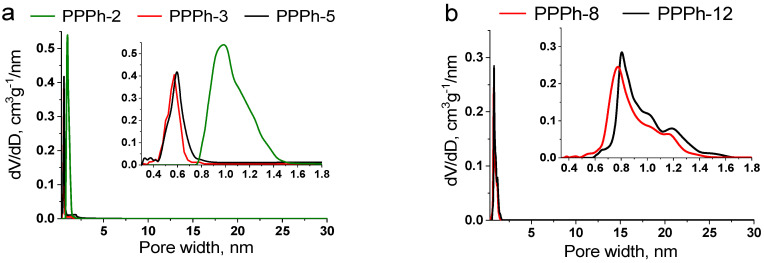
Pore size distribution curves of PPPhs calculated by NLDFT. (**a**) PPPh-2,3,5 and (**b**) PPPh-8,12.

Remarkably, a distinct sorption capacity was observed for the polymers on the basis of tetrahedral and trigonal monomers. The results of adsorption–desorption experiments are summarized in Table 2. In contrast to trigonal-based polymers, tetrahedral-based samples exhibited significant differences in N_2_ and CO_2_ porosity measurements. Specifically, a very low porosity measured with N_2_ was determined for the Polymers 3, 5, and 6. As a consequence, the calculated specific surface areas did not exceed 38 m^2^ g^−1^. However, Sample 2 demonstrated the highest specific surface area, S_BET_ = 667 m^2^ g^−1^. The opposite situation was observed when turning to CO_2_ isotherms. In this case, the high values of SSA were determined for all polymers. A closer look at the porosity parameters revealed that PPPhs 3, 5, and 6 are ultramicroporous in nature. Their pore volumes are in the range of 0.042–0.110 cm^2^ g^−1^, and the pore diameters are 0.6 nm. Polymer 2 is characterized by a larger pore volume, up to 0.174 cm^2^ g^−1^, and the average pore width equals 1.0 nm, indicating that this polymer is rather microporous. The pore size distributions for the samples are given in Figure 3a. This difference explains such distinct porosity values obtained with different sorbate molecules: N_2_ and CO_2_. CO_2_ is a linear molecule that resolves more easily the ultramicropores. Moreover, CO_2_ adsorption measurements are performed at 273 K, in contrast to 77 K of N_2_ adsorption. The higher temperature aids in kinetic flexibility of the polymer chains and favors the diffusion of sorbate molecules, which positively influences the adsorption process. This enables easier access of CO_2_ to ultramicropores in comparison with N_2_. The results are consistent with previous reports suggesting that CO_2_ and H_2_ are more preferable for evaluation of porosity in ultramicroporous materials [18,49,50].

Nevertheless, Polymer 2 demonstrated a steep increase in the amount of adsorbed N_2_ at low P/P_0_, which is indicative of the presence of micropores. The micropore surface area, calculated by the T-plot method, was estimated to be 350 m^2^ g^−1^, indicating that almost 50% of the total surface area is composed of micropores. Considering the similar chemical structure of Polymers 2 and 3 (they are constituted of the similar building blocks (A4 and B2_1_)), the ultramicroporous character of the latter can be attributed to a higher molecular weight. The high molecular weight may promote the compactization of the macromolecule and contribute to a denser polymer molecule arrangement [51].

When switching to monomer B2_2_ containing the oxygen-bridging group (Polymers 5 and 6), the surface areas derived from CO_2_ isotherms were lower than those of the polymers composed of the more rigid B2_1_ monomer. Obviously, this related to a larger conformational freedom, reducing the overall porosity. Nevertheless, both samples demonstrated moderate specific surface areas, S = 238 and S = 443 m^2^ g^−1^ for Polymers 5 and 6, respectively. The mean pore sizes were determined to be 0.6 nm, confirming that the polymers were still ultramicroporous. The polymer with the higher molecular weight had the larger SSA, while the pore size was affected insignificantly in comparison with the B2_1_ monomer.

It should be noted that design of the porous organic polymers possessing uniformly sized ultramicropores is still very challenging [52,53,54]. The lack of the N_2_ adsorption observed for Polymers 3, 5, and 6 confirms the absence of the large pores, which could be accessible for N_2_ molecule. Narrow size distribution, along with ultramicroporosity that was achieved for tetrahedral-based polyphenylenes, is of particular interest for application in gas separation because of the excellent selectivity that is usually observed for uniformly sized ultramicroporous materials.

The use of 1,3,5-triethynylbenzene as a tri-branched unit also yielded the PPPhs with high surface areas up to 515 m^2^ g^−1^. Again, the values calculated from CO_2_ adsorption isotherms exceeded those of N_2_-derived ones; however, the difference was not crucial. The bis(cyclopentadienone) B2_1_ containing the phenylene-bridging group was more potent for microporosity formation, resulting in the macromolecules of higher surface areas in comparison with the B2_2_ monomer, as was observed earlier for tetraphenylmethane-based polymers. Trigonal geometry of the monomer A3 apparently provided the appearance of larger pores in the range of 0.8–1.2 nm, although ultramicropores were also preserved (Figure 3b). Increase in the overall pore volumes and widths is most likely related to a less dense packing of the polymer chains since a larger free volume is accessible when the macromolecule growth is in three directions rather than four.

## 4. Conclusions

In the present work, we showed that Diels–Alder polycondensation is a promising approach for construction of non-crosslinking microporous polymers. By varying the synthetic conditions, the soluble hyperbranched polyphenylenes possessing ultramicroporosity were successfully synthesized. The degree of branching and spatial arrangement of starting monomer had a strong impact on the porosity parameters, e.g., surface areas and pore volumes. The monomer with tetrahedral geometry was beneficial over the trigonal planar one to construct the polymers of ultramicroporous nature and, in some cases, possessed the larger surface area. Importantly, employment of tetra-branched monomer provided ultramicroporous polymers with narrow size distribution, while utilization of the tri-branched unit led to an increase in the pore sizes and formation of micropores along with ultramicropores, as confirmed by DFT analysis of pore size distributions. The results of the study open a new way for rational design of ultramicroporous non-networking polymers. Taking advantages of exceptional chemical and thermal stability and ultramicroporous structure, the developed PPPhs are promising materials for further investigation in a plethora of applications.

## Figures and Tables

**Table 1:** Reaction conditions and molecular characteristics.

Polymer ^a^	Monomer A	Monomer B	C ^b^, mol L^−1^	Time, h	M_w_^c^·10^−3^, g·mol^−1^	M_w_/M_n_	Yield, %
1	A4	B2_1_	0.05	10	Insoluble	-	99.0
2	A4	B2_1_	0.01	10	16.3	1.69	76.2
3	A4	B2_1_	0.01	17	35.1	2.11	88.6
4	A4	B2_2_	0.05	6	Insoluble	-	99.0
5	A4	B2_2_	0.01	10	23.2	2.21	88.5
6	A4	B2_2_	0.01	20	47.4	2.56	95.4
7	A3	B2_1_	0.05	10	Insoluble	-	99.2
8	A3	B2_1_	0.01	10	17.2	2.35	78.7
9	A3	B2_1_	0.01	17	23.4	2.47	96.3
10	A3	B2_2_	0.1	7	Insoluble	-	99.1
11	A3	B2_2_	0.05	10	317.3	3.53	75.3
12	A3	B2_2_	0.05	22	789.3	20.61	98.0

^a^ Reactions were conducted in diphenyl ester at 165 °C; ^b^ Total molar concentration of the monomers A3 (or A4) + B2; ^c^ Apparent molecular weights determined by SEC with polystyrene as a standard.

**Table 2:** Surface areas and porosity parameters of porous polyphenylenes.

Polymer	S_BET_^a^ (N_2_), m^2^ g^−1^	S_DFT_^b^ (CO_2_), m^2^ g^−1^	V_pore_^c^ (CO_2_), cm^3^ g^−1^	D ^d^, nm
2	667	751	0.174	1.0
3	19	553	0.110	0.6
5	38	238	0.042	0.6
6	23	443	0.097	0.6
8	247	332	0.061	0.8–1.2
9	313	437	0.097	0.8–1.2
11	356	468	0.098	0.8–1.2
12	404	515	0.107	0.8–1.2

^a^ Specific surface areas are calculated on the basis of the BET equation. ^b^ SSA are calculated on the basis of NLDFT with a slit-pore mode. ^c^ Pore volumes are calculated by DFT method from CO_2_ isotherms. ^d^ Mean diameters are calculated by DFT method from CO_2_ isotherms.

## Data Availability

The data are available upon request.

## Supplementary Materials

The following supporting information can be downloaded at: https://www.mdpi.com/article/10.3390/polym15092060/s1, Figure S1: SEC chromatograms of PPPhs 9 (a), 6 (b), and 11 (c).; Figure S2: NMR ^1^H (a) and ^13^C (b) spectra and the proposed structure of porous polyphenylene (c); Figure S3: NMR ^1^H (a) and ^13^C (b) spectra of PPPh-8; Figure S4: TGA results of the polymers; Figure S5: DSC curves for PPPhs.
