# Supplementary material for: Ultramicroporous Polyphenylenes via Diels–Alder Polycondensation Approach
Source: Polymers (Basel). 2023 Apr 26;15(9):2060. doi: 10.3390/polym15092060 (PMC10181309; doi:10.3390/polym15092060)
Supplement: Supplementary file 1 [file polymers-15-02060-s001.zip › polymers-2368257-supplementary.pdf]

Supporting Information

Ultramicroporous polyphenylenes via Diels-Alder polycon-densation approach

Svetlana A. Sorokina, Nina V. Kuchkina, Alexander V. Mikhalchenko, Irina Yu. Krasnova, Dmitry A. Khanin, Kirill M. Skupov, Zinaiada B. Shifrina

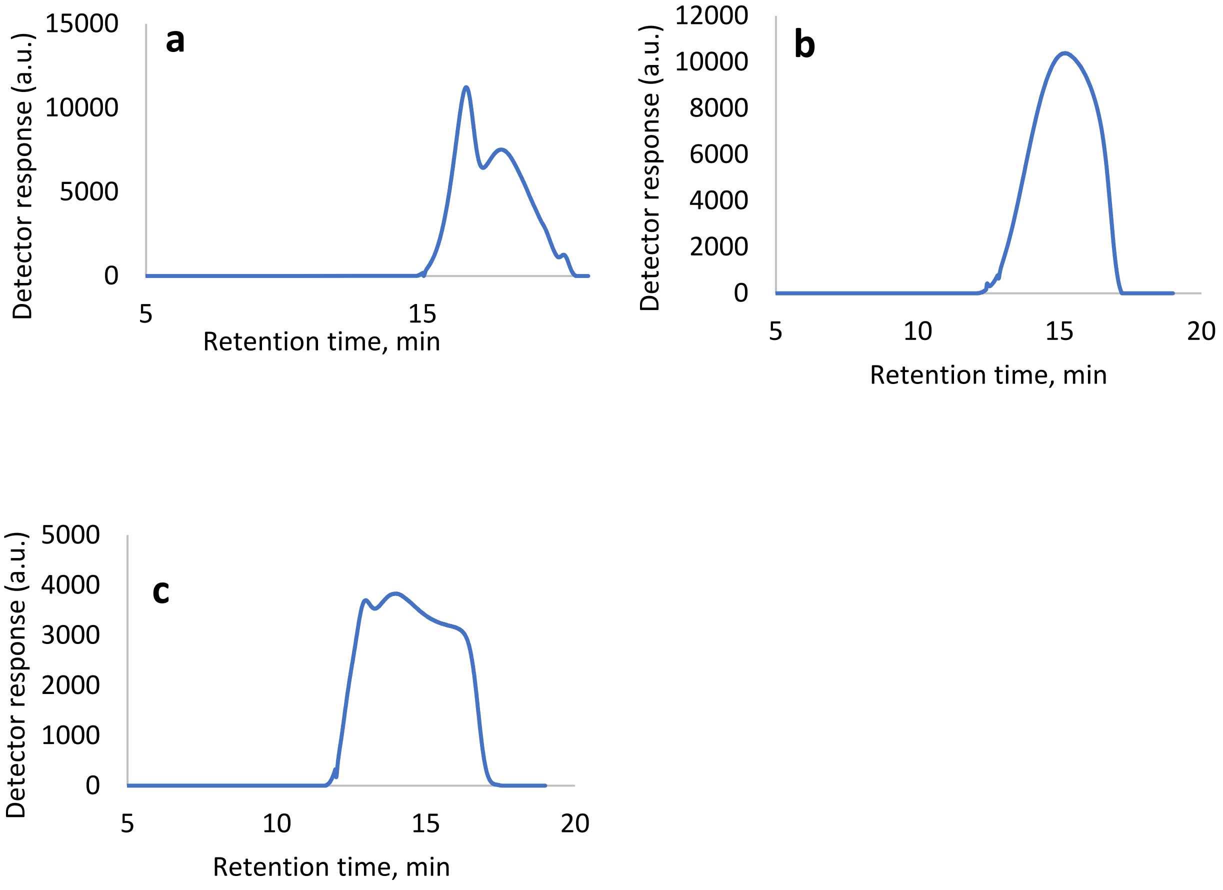

Figure S1. SEC chromatograms of PPPhs 9 (a), 6 (b) and 11 (c).

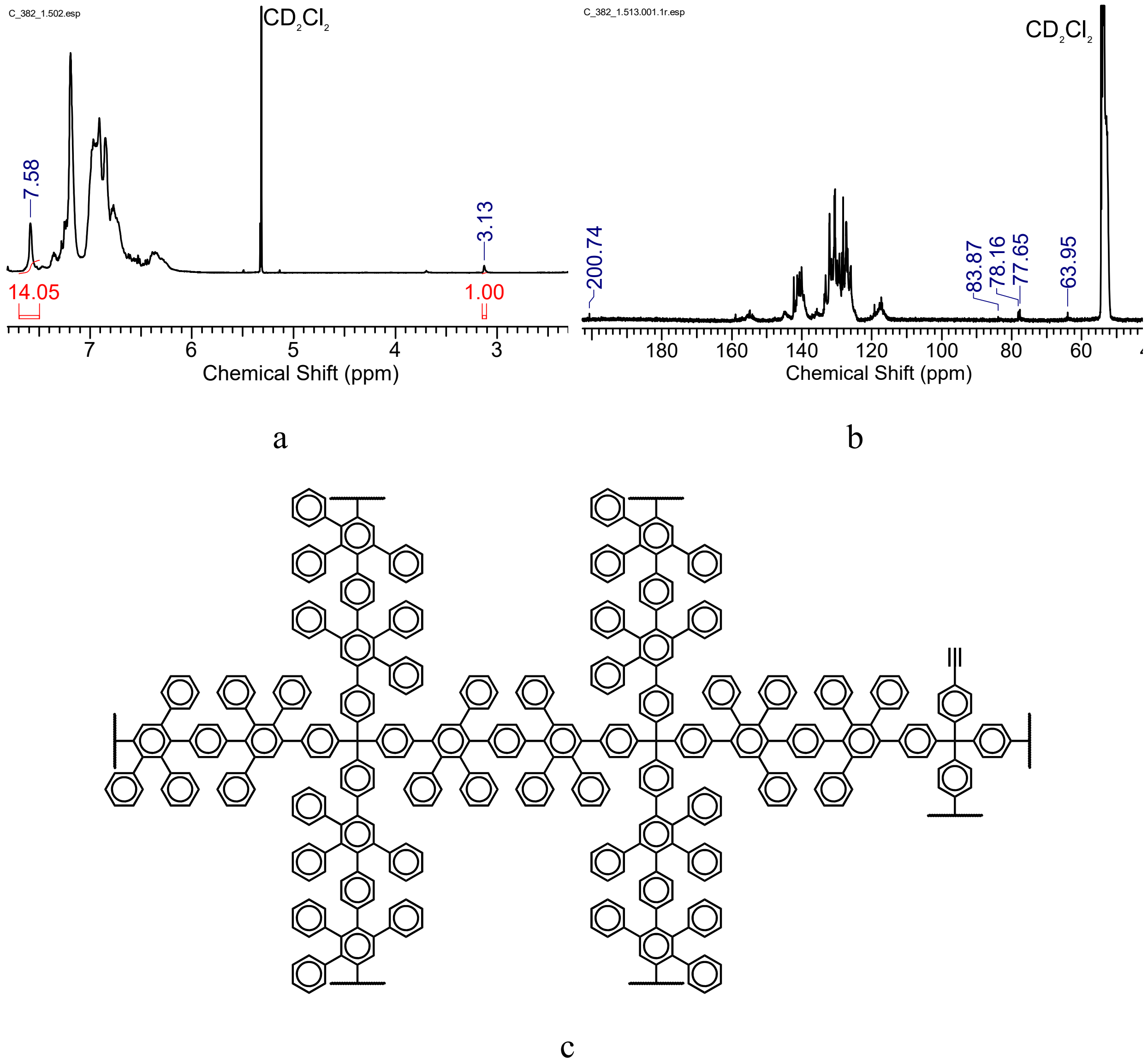

Figure S2. NMR ^{1}H (a) and ^{13}C (b) spectra and the proposed structure of porous polyphenylene (c)

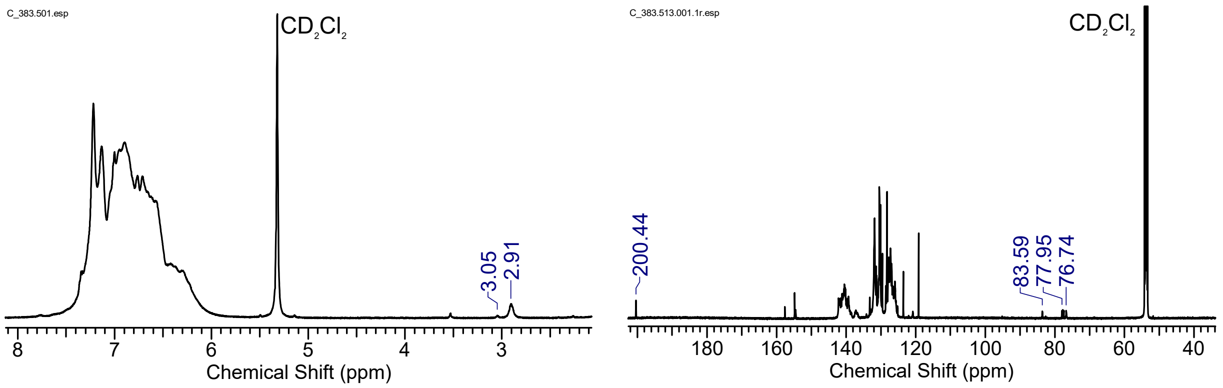

a b

Figure S3. NMR ^{1}H (a) and ^{13}C (b) spectra of PPPh-8

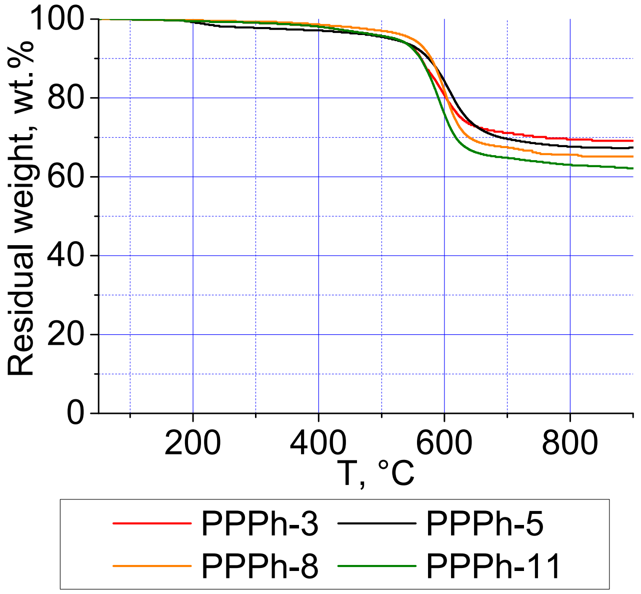

Figure S4. TGA results of the polymers

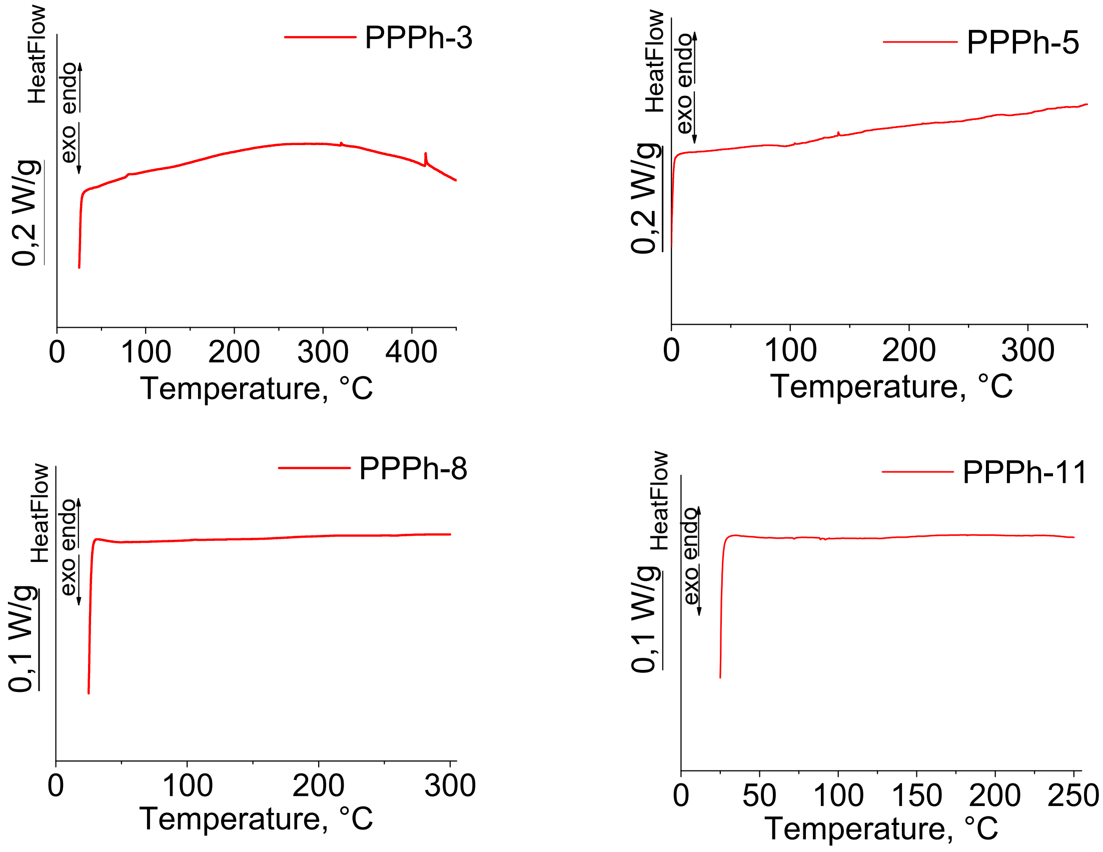

Figure S5. DSC curves for PPPhs
